# Signaling through the Phosphatidylinositol 3-Kinase (PI3K)/Mammalian Target of Rapamycin (mTOR) Axis Is Responsible for Aerobic Glycolysis mediated by Glucose Transporter in Epidermal Growth Factor Receptor (*EGFR*)-mutated Lung Adenocarcinoma*[Fn FN2]

**DOI:** 10.1074/jbc.M115.660498

**Published:** 2015-05-28

**Authors:** Hideki Makinoshima, Masahiro Takita, Koichi Saruwatari, Shigeki Umemura, Yuuki Obata, Genichiro Ishii, Shingo Matsumoto, Eri Sugiyama, Atsushi Ochiai, Ryo Abe, Koichi Goto, Hiroyasu Esumi, Katsuya Tsuchihara

**Affiliations:** From the ‡ Division of Translational Research, Exploratory Oncology Research and Clinical Trial Center, National Cancer Center, Kashiwa, Chiba 277-8577, Japan,; the § Department of Integrated Biosciences, Graduate School of Frontier Sciences, The University of Tokyo, Kashiwa, Chiba 277-8561,; the ¶ Thoracic Oncology and; ** Pathology Divisions, Research Center for Innovative Oncology, National Cancer Center Hospital East, Kashiwa, Chiba 277-8577, Japan, and; the Divisions of ‖ Immunobiology and; ‡‡ Clinical Research, Research Institute for Biomedical Sciences, Tokyo University of Science, Noda, Chiba 278-0022, Japan

**Keywords:** epidermal growth factor receptor (EGFR), glucose transport, glycolysis, lung cancer, mammalian target of rapamycin (mTOR), metabolomics, phosphatidylinositol 3-kinase (PI3K)

## Abstract

Oncogenic epidermal growth factor receptor (EGFR) signaling plays an important role in regulating global metabolic pathways, including aerobic glycolysis, the pentose phosphate pathway (PPP), and pyrimidine biosynthesis. However, the molecular mechanism by which EGFR signaling regulates cancer cell metabolism is still unclear. To elucidate how EGFR signaling is linked to metabolic activity, we investigated the involvement of the RAS/MEK/ERK and PI3K/AKT/mammalian target of rapamycin (mTOR) pathways on metabolic alteration in lung adenocarcinoma (LAD) cell lines with activating *EGFR* mutations. Although MEK inhibition did not alter lactate production and the extracellular acidification rate, PI3K/mTOR inhibitors significantly suppressed glycolysis in *EGFR*-mutant LAD cells. Moreover, a comprehensive metabolomics analysis revealed that the levels of glucose 6-phosphate and 6-phosphogluconate as early metabolites in glycolysis and PPP were decreased after inhibition of the PI3K/AKT/mTOR pathway, suggesting a link between PI3K signaling and the proper function of glucose transporters or hexokinases in glycolysis. Indeed, PI3K/mTOR inhibition effectively suppressed membrane localization of facilitative glucose transporter 1 (GLUT1), which, instead, accumulated in the cytoplasm. Finally, aerobic glycolysis and cell proliferation were down-regulated when GLUT1 gene expression was suppressed by RNAi. Taken together, these results suggest that PI3K/AKT/mTOR signaling is indispensable for the regulation of aerobic glycolysis in *EGFR*-mutated LAD cells.

## Teaser

**Background:** EGFR signaling maintains aerobic glycolysis, but the molecular mechanism is still undefined.

**Results:** Drug inhibition studies reveal that downstream signaling via the PI3K pathway is critical for glucose transport and metabolism.

**Conclusion:** The PI3K signaling regulates key metabolic activities in *EGFR*-mutant lung adenocarcinoma.

**Significance:** These data may guide the development of chemotherapeutic options, including targeting of the PI3K pathway and glucose transporter machinery.

## Introduction

Cancer genomics using next generation sequencing technology have successfully characterized a number of therapeutic targets of lung cancer (1–6). *EGFR* mutations have been identified in more than 50% of lung adenocarcinomas (LADs)^2^ from East Asian non-smokers, and these tumors have been termed oncogene-addicted to reflect their dependence on EGFR-mediated, prosurvival signaling (7). Activated EGFR stimulates downstream oncogenic signaling pathways, including the PI3K/AKT/mTOR and RAS/RAF/MEK/ERK pathways, to promote cell survival and proliferation (8, 9), and disruption of these signaling pathways by EGFR tyrosine kinase inhibitors (TKIs) such as gefitinib and erlotinib results in susceptibility of *EGFR*-mutated tumors to apoptosis (10).

Somatic mutations in cancer driver genes, tumor suppressors, and amplified oncogenes such as *EGFR*, *RAS*, *BRAF*, *MYC*, isocitrate dehydrogenase (*IDH*), and fumarate hydratase (*FH*) have been shown to be linked to specific alterations in metabolic activity in cancer cells (11–14). Such alterations have been considered to result in the Warburg effect, which describes the phenomenon whereby cancer cells choose high-rate aerobic glycolysis to metabolize glucose to lactate despite the presence of adequate oxygen (11–14). In contrast, normal cells mainly utilize glucose via mitochondrial oxidative phosphorylation to generate ATP (15–18). The mechanism by which such metabolic alterations are induced by cancer gene mutations, however, is not fully understood.

GLUT1, or solute carrier 2A 1 (SLC2A1), has been shown to play a role in cancer cell metabolic reprogramming (19, 20). At present, two classes of glucose transporters, GLUTs and sodium-glucose symporters, exist in cancer cells (21, 22). The GLUT (SLC2A) family is a wide group of membrane proteins including 14 hexose transporters that facilitate the transport of glucose over the plasma membrane (21, 22). In some cancers, GLUT1 overexpression has been found to be associated with tumor progression (19, 20, 23). The sodium-glucose symporter family is predominantly expressed in the small intestine and kidney. One report has suggested that EGFR physically associates with and stabilizes SGLT1 to promote glucose uptake into cancer cells (24).

We have shown previously that mutant EGFR signaling maintains up-regulated aerobic glycolysis, PPP, and *de novo* pyrimidine biosynthesis (11). In this scenario, the tyrosine kinase activity of EGFR is dysregulated by gene mutations that lead to aberrant EGFR signaling via the RAS/MEK/ERK and PI3K/AKT/mTOR pathways (8, 9). Here we show that, of these two pathways, the PI3K/AKT/mTOR signaling axis plays a more critical role in regulating glycolysis in *EGFR*-mutated LAD and appears to facilitate the proper localization of the glucose transporter GLUT1. Suppression of *GLUT1* after blocking with siRNA resulted in decreased lactate production and cell proliferation in *EGFR*-mutated LAD. These findings indicate that the PI3K/AKT/mTOR pathway is responsible for aerobic glycolysis by regulating GLUT1 localization in *EGFR*-mutated LAD.

## Experimental Procedures

### 

#### 

##### Purchased Materials

Cell lines were purchased from Immunobiological Laboratories (Fujioka, Japan) and the ATCC. RPMI 1640 medium (R8758 and R1383), PBS, and 2-deoxy-d-glucose (2DG) were purchased from Sigma-Aldrich (St. Louis, MO). FBS was purchased from Biowest (Nuaille, France). Erlotinib (ERLO), AZD6244, BEZ235, AZD9291, and PKI-587 were purchased from Selleck (Houston, TX). EnVision TM+, peroxidase-conjugated anti-rabbit and antibody diluent were purchased from Dako (Glostrup, Denmark). Dimethyl sulfoxide (DMSO), glucose, and 3,3′-diaminobenzidine were purchased from Wako Pure Chemicals Industries (Osaka, Japan). Cell counting kit 8 was purchased from Dojindo Laboratories (Kumamoto, Japan). Lactate assay kit II was purchased from BioVision (Milpitas, CA). The FluxPak XF24 assay pack and XF glycolysis stress test kit were purchased from Seahorse Bioscience (North Billerica, MA). The Countess automated cell counter, including trypan blue and chamber slides, was purchased from Invitrogen. The Mini-Protean TGX precast gel, Trans-Blot turbo transfer system, and Trans-Blot turbo transfer pack were purchased from Bio-Rad. Primary antibodies specific for EGFR, phospho-EGFR (pEGFR), AKT, phospho-AKT (pAKT), ERK1/2, phospho-ERK1/2 (pERK), and β-actin were purchased from Cell Signaling Technologies (Danvers, MA). Anti-GLUT1 and anti-Na/K were purchased from Abcam (Cambridge, UK). GLUT1-targeting siRNA and the BCA protein assay were purchased from Thermo Fisher Scientific (Waltham, MA). The peroxidase-linked secondary antibodies for Western blot analysis, HRP-linked sheep anti-mouse IgG, and donkey anti-rabbit IgG were purchased from GE Healthcare. Alexa Fluor 488-conjugated anti-rabbit IgG and Alexa Fluor 594-conjugated anti-mouse IgG were purchased from Invitrogen. Oligomycin was purchased from Merck Millipore (Darmstadt, Germany). Vectashield mounting medium was purchased from Vector Laboratories (Burlingame, CA). FITC-conjugated anti-rabbit IgG antibody was purchased from Jackson ImmunoResearch Laboratories (West Grove, PA).

##### Cell Survival Assay and Proliferation Assay

*EGFR* mutant LAD cells were seeded in RPMI 1640 medium containing various concentrations of inhibitors in 96-well cell culture plates. After 72 h of incubation at 37 °C and 5% CO_2_, cell viability was analyzed by WST-8 assay using cell counting kit 8. The optical density of the cell culture medium in each well was read at 450 nm on a microplate reader (Molecular Devices, Sunnyvale, CA). Viable cells were enumerated by trypan blue exclusion using a Countess automated cell counter (Life Technologies).

##### Western Blotting

Cells were lysed in radioimmune precipitation assay buffer on ice for 10 min and centrifuged at 15,000 × *g* for 10 min. The protein content of supernatants was quantified by BCA assay (Pierce). Proteins were separated on a commercially available 4–20% gradient SDS-polyacrylamide gel (Mini-Protean TGX, Bio-Rad) and transferred to a polyvinylidene difluoride membrane (Trans-Blot turbo transfer pack, Bio-Rad). They were then incubated overnight with primary antibodies (1:1000). The primary antibodies used in this study are described above. ECL anti-rabbit IgG HRP-linked whole antibody (GE Healthcare, 1:10,000) was used as secondary antibody, and signals were detected using ECL Western blotting detection reagent (GE Healthcare) and x-ray films (GE Healthcare). β-actin was used as a loading control.

##### Lactate Assay

Lactate in culture medium was quantified with a lactate assay kit II according to the instructions of the manufacturer (Biovision, Mountain View, CA). After centrifugation (3500 rpm, 15 min, 4 °C), cell culture medium was stored at −20 °C. Samples were diluted in assay buffer and mixed with lactate reaction mixture for 30 min. The optical density of the mixture in each well was measured at 450 nm on a microplate reader (Molecular Devices). The lactate concentration was calculated from a standard curve and normalized against cell numbers and time.

##### Measurement of ECAR and Oxygen Consumption Rate (OCR)

ECAR and OCR were measured with an XF glycolysis stress test kit according to the instructions of the manufacturer (Seahorse Bioscience). In brief, 4.5 × 10^4^ cells were plated onto XF24 plates in RPMI 1640 medium (10% FBS and 2 mm glutamine) and incubated at 37 °C, 5% CO_2_ overnight. Medium including DMSO or inhibitors was placed into each well, and cells were incubated for 6 h. Cells were washed with assay medium (minus glucose and unbuffered RPMI 1640 medium (Sigma, R1383)), replaced with assay medium, and then placed at 37 °C in a CO_2_-free incubator for 30 min. ECAR and OCR were monitored using a Seahorse Bioscience XF24 extracellular flux analyzer over time. Each cycle consisted of 3 min of mixing, 3 min of waiting, and 3 min of measuring. Glucose, oligomycin, and 2-deoxy-d-glucose were diluted into XF24 medium and loaded into the accompanying cartridge to achieve final concentrations of 10 mm, 5 μm, and 100 mm, respectively.

##### Metabolite Measurements

H1975 cells were grown in RPMI 1640 medium containing 11.1 mm [U-^13^C]glucose ([^13^C]Glc_6_) in the presence of DMSO, AZD9291, or PKI-587. Metabolic extracts were prepared after 6 h of incubation and analyzed using a capillary electrophoresis (CE)-connected electrospray ionization (ESI)-TOFMS and CE-MS/MS system (Human Metabolome Technologies; HMT, Inc., Tsuruoka, Japan, F-SCOPE) (25, 26). For quantitative static metabolomic analysis, samples were prepared from 2–5 × 10^6^ cells with methanol containing internal standard solution (HMT) and analyzed using a CE-connected ESI-TOF/MS and CE-MS/MS system (HMT, C-SCOPE). Detailed procedures have been published in our previous paper (11).

##### Immunohistochemistry

All immunohistochemistry analyses were performed on paraffin-embedded tissues obtained from the primary tumor in the surgical specimen. This study was approved by the Institutional Review Board of the National Cancer Center, Japan (no. 2014-325). All *EGFR* mutation status information used in this study was obtained from a database at the Division of Thoracic Oncology, National Cancer Center Hospital East, Kashiwa, Japan. We prepared and used 4-μm-thick paraffin sections cut from a paraffin block containing histological findings that were representative of the tumor. Antigen retrieval was performed in citrate buffer solution (pH 6.0). Endogenous peroxidase was blocked with 0.3% H_2_O_2_ in methanol for 15 min, and all slides were heated to 95 °C by exposure to microwave radiation for 20 min. Slides were washed in PBS, and, after 1-h incubation at room temperature with the primary antibodies, the slides were incubated for 30 min with a labeled polymer EnVision TM+ peroxidase-conjugated anti-rabbit antibody (Dako, Tokyo, Japan). The chromogen used was 2% 3,3^′^-diaminobenzidine in 50 mm Tris buffer (pH 7.6) containing 0.3% hydrogen.

##### Immunofluorescence

Cells were seeded in a Ibidi 35-mm microdish (Martinsried, Germany) and cultured overnight. They were then incubated with inhibitors for 6 h. Cells were washed three times and fixed with 4% paraformaldehyde for 15 min. After washing, the fixed cells were treated with blocking buffer (1% BSA, 3% TritonX-100, and PBS) and incubated with primary antibody at 4 °C overnight. After washing, cells were incubated in secondary antibody (Alexa Fluor 488-conjugated anti-rabbit IgG and Alexa Fluor 594-conjugated anti-mouse IgG) for 1 h, stained with DAPI, mounted with Vectashield mounting medium, and analyzed using a confocal microscopy laser-scanning microscope 710 (Carl Zeiss, Oberkochen, Germany).

##### Flow Cytometric Analysis

To quantitatively detect the expression of membrane-bound GLUT1, cells were fixed with 80% ethanol, incubated with anti-GLUT1 antibody (Abcam), and then stained with the appropriate FITC-conjugated anti-rabbit IgG antibody (Jackson ImmunoResearch Laboratories). Quantification of FITC fluorescence intensity was performed using a FACSCanto II (BD Biosciences).

##### siRNA Transfection

LAD cells (1.0 × 10^5^) were transfected with 5 nm siGLUT1#1 (catalog no. s12925, Thermo Fisher Scientific), siGLUT1#2 (catalog no. s12926, Thermo Fisher Scientific), or siNC (catalog no. 4390843, Thermo Fisher Scientific) using Lipofectamine RNAiMAX reagent (Thermo Fisher Scientific).

##### Statistical Analyses

Unless indicated otherwise, results are reported as mean ± S.D. Statistical analyses were done by two-tailed Student's *t* test.

## Results

### 

#### 

##### EGFR-mutant LAD Cells Were More Sensitive to Dual PI3K/mTOR Inhibitors than MEK Inhibitor

**FIGURE 1.**
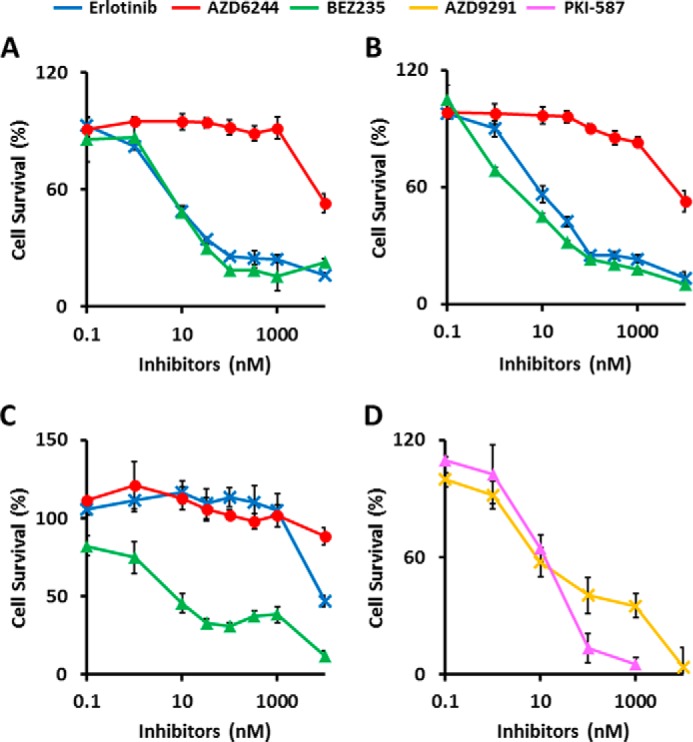
**EGFR-mutant LAD cells are more sensitive to dual PI3K/mTOR inhibitors than MEK inhibitor.** Cells were treated with inhibitors at the indicated concentrations for 72 h, and viability was assessed using the WST-8 assay. *A–D*, HCC827 (*A*), PC-9 (*B*) and H1975 cells (*C* and *D*). The data are shown as the mean ± S.D. (*n* = 6). *A—C*, *blue line*, erlotinib; *red line*, AZD6244; *green line*, BEZ235. *D*, *magenta line*, PKI-587; *yellow line*, AZD9291. The *in vitro* IC_50_ for the growth of HCC827 was determined to be 0.010 μm for erlotinib, >10 μm for AZD6244, and 0.011 μm for BEZ235. PC-9 had an IC_50_ of 0.019 μm for erlotinib, >10 μm for AZD6244, and 0.005 μm for BEZ235. H1975 had an IC_50_ of >10 μm for erlotinib, >10 μm for AZD6244, 0.042 μm for BEZ235, 0.050 μm for AZD9291, and 0.042 μm for PKI-587.

We studied three lung adenocarcinoma cell lines that possessed the *EGFR* mutation in either exon 19 or exon 21. The cell lines HCC827 and PC-9 both carried the delE746-A750 mutation, whereas NCI-H1975 cells carried EGFR L858R plus T790M, a mutation that confers resistance to erlotinib. First, we confirmed that the HCC827 and PC-9 cell lines were highly sensitive to the EGFR TKI erlotinib (Fig. 1, *A* and *B*), with IC_50_ values in the nanomolar range compared with the erlotinib-resistant H1975 (Fig. 1*C*). We also treated H1975 cells with AZD9291, a selective third-generation irreversible EGFR inhibitor of T790M resistance mutants (27). Here we confirmed that AZD9291 reduced viability in H1975 cells to a greater extent compared with erlotinib after 3 days of treatment (Fig. 1*D*).

Several signaling pathways related to cellular proliferation and survival are activated downstream of EGFR, including the RAS/MEK/ERK and PI3K/AKT/mTOR pathways. AZD6244 (selumetinib) is a non-ATP competitive inhibitor and highly specific for MEK, a key enzyme in the RAS/RAF/MEK/ERK pathway, whereas NVP-BEZ235 (dactolisib) and PKI-587 (gedatolisib) are dual pan PI3K and mTOR inhibitors. Compared with the MEK inhibitor AZD6244 (IC_50_ = >10 μm), treatment with the PI3K/mTOR inhibitor BEZ235 inhibited growth of all three cell lines more effectively, with similar dose-dependent decreases in viability (mean IC_50_ = 11, 5, and 42 nm for the HCC827, PC-9, and H1975 cell lines, respectively) (Fig. 1, *A–C*). In addition, PKI-587 also effectively kills H1975 cells harboring the T790M resistance mutation (Fig. 1*D*).

##### Altered Phosphorylation of EGFR Signaling Proteins in EGFR-mutant LAD Cells after Treatment with Inhibitors

**FIGURE 2.**
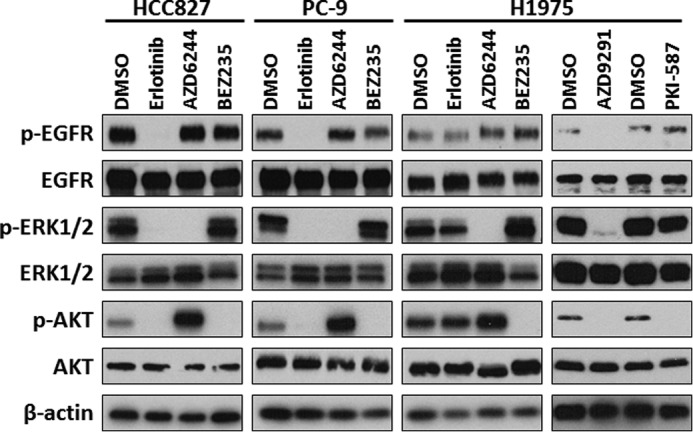
**Altered phosphorylation of EGFR signaling proteins in *EGFR*-mutant LAD cells after treatment with inhibitors.** Western blot analysis showing pEGFR, total EGFR, pERK, total ERK, pAKT), total AKT, and β-actin as a loading control in HCC827, PC-9, and H1975 cells treated with the indicated inhibitors. Equivalent amounts of proteins from whole cell lysates were subjected to WB analysis to detect the indicated proteins.

To verify that the compounds used inhibited the corresponding signaling pathways, phosphorylation of EGFR signaling molecules was investigated by Western blot (WB) analysis in HCC827, PC-9, and H1975 cells (Fig. 2). We examined the effect of the inhibitors on molecular markers of the EGFR signaling cascade (EGFR, AKT, and ERK) in LAD cells incubated in the presence of erlotinib, AZD6244, BEZ235, AZD9291, and PKI-587, respectively. WB analysis showed that phosphorylation of EGFR (pEGFR), AKT (pAKT), and ERK (pERK) was inhibited at 6 h after erlotinib treatment in HCC827 and PC9 cells (Fig. 2). Alterations in any of the major EGFR signaling cascades were not observed in EGFR TKI-resistant H1975 cells treated with erlotinib, whereas treatment with AZD9291 down-regulated phosphorylation of EGFR and downstream signaling (Fig. 2). AZD6244 treatment inhibited the RAS/MEK/ERK pathway and slightly increased AKT phosphorylation in all three cell lines (Fig. 2). Signaling through the PI3K/AKT/mTOR pathway was down-regulated in all three cell lines in the presence of BEZ235 and PKI-587 (Fig. 2), as shown by decreased pAKT and consistent with previous reports (28, 29).

##### Glycolytic Activities Decreased after Inhibition of the PI3K/AKT/mTOR but not the RAS/MEK/MAPK Pathway in EGFR-mutant LAD Cells

**FIGURE 3.**
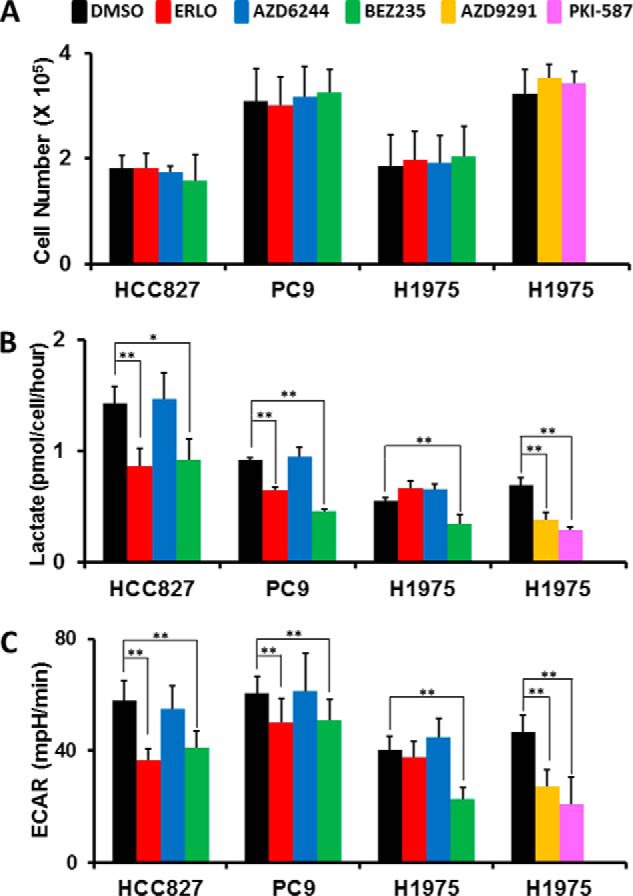
**Glycolytic activities decreased after inhibition of the PI3K/AKT/mTOR but not the RAS/MEK/MAPK pathway in *EGFR*-mutant LAD cells.** *A*, cell growth responses at 6 h to 1 μm of indicated inhibitors were measured by trypan blue staining. The cell numbers for HCC827, PC-9, and H1975 cells treated with of DMSO (*black*), ERLO (*red*), AZD6244 (*blue*), BEZ235 (*green*), AZD9291 (*yellow*), and PKI-587 (*magenta*) are shown. The data are shown as the mean ± S.D. (*n* = 4). *B*, extracellular lactate production in HCC827, PC-9, and H1975 cell lines treated with DMSO (*black*), ERLO (*red*), AZD6244 (*blue*), BEZ235 (*green*), AZD9291 (*yellow*), and PKI-587 (*magenta*) 6 h after inhibitor treatment. *Error bars* indicate mean ± S.D. (*n* = 4–12). *, *p* < 0.05; **, *p* < 0.01 *versus* control by two-tailed Student's *t* test. *C*, ECAR values of the HCC827, PC-9, and H1975 cell lines treated with DMSO (*black*), ERLO (*red*), AZD6244 (*blue*), BEZ235 (*green*), AZD9291 (*yellow*), and PKI-587 (*magenta*) after 36 min of flux assay. All cells were treated with the indicated inhibitors (1 μm) for 6 h before each assay. Results are reported as the mean ± *S.D.* (*n* = 7–12). **, *p* < 0.01 *versus* control by two-tailed Student's *t* test.

Treatment with EGFR TKIs decreases lactate production, glucose consumption, and the ECAR in LAD cells, indicating that EGFR signaling maintains aerobic glycolysis (11). By using specific inhibitors to block either the RAS/MEK/ERK or PI3K/AKT/mTOR pathway, we observed that a 72-h incubation with dual PI3K/mTOR inhibitors led to a dramatic reduction in cell viability in BEZ235-sensitive *EGFR*-mutant cell lines, suggesting that the PI3K/AKT/mTOR pathway is critical for aerobic glycolysis downstream of EFGR (Fig. 1). To examine this further, we set up experimental conditions where inhibitor treatment was given at a relatively higher concentration (1 μm) and shorter time frame (6 h), a method we have used previously for EGFR TKI treatment (11). Under these experimental conditions, all cells grew equally and, thereby, standardized the number of viable cells analyzed (Fig. 3*A*). Our results showed that lactate production was decreased in HCC827 and PC-9 cells after treatment with erlotinib but not changed in H1975 cells (Fig. 3*B*). Moreover, we discovered that exposure of the cells to PI3K/mTOR inhibitors for up to 6 h significantly lowered the rate of lactate accumulation in the medium of LAD cell lines, whereas cells treated with MEK inhibitor did not show this effect (Fig. 3*B*). To better define lactate production derived from glucose, we measured ECAR, an indicator of lactate production, and the OCR, an indicator of oxidative phosphorylation, using a flux analyzer. The ECAR was statistically higher in DMSO controls and after AZD6244 MEK inhibitor treatment compared with PI3K/mTOR inhibitor-treated HCC827, PC-9, and H1975 cells (Fig. 3*B*). In contrast to the ECAR, the OCR was not changed significantly changed in HCC827, PC-9, and H1975 cells under these experimental conditions (data not shown). Treatment with the third-generation EGFR TKI AZD9291 caused a reduction in both lactate production and ECAR in H1975 cells (Fig. 3, *B* and *C*). These data suggest that PI3K/AKT/mTOR signaling plays a crucial role in the maintenance of aerobic glycolysis in *EGFR*-mutant LAD cells.

##### PI3K/AKT/mTOR Signaling Up-regulates Glycolysis and the Pentose Phosphate Pathway

**FIGURE 4.**
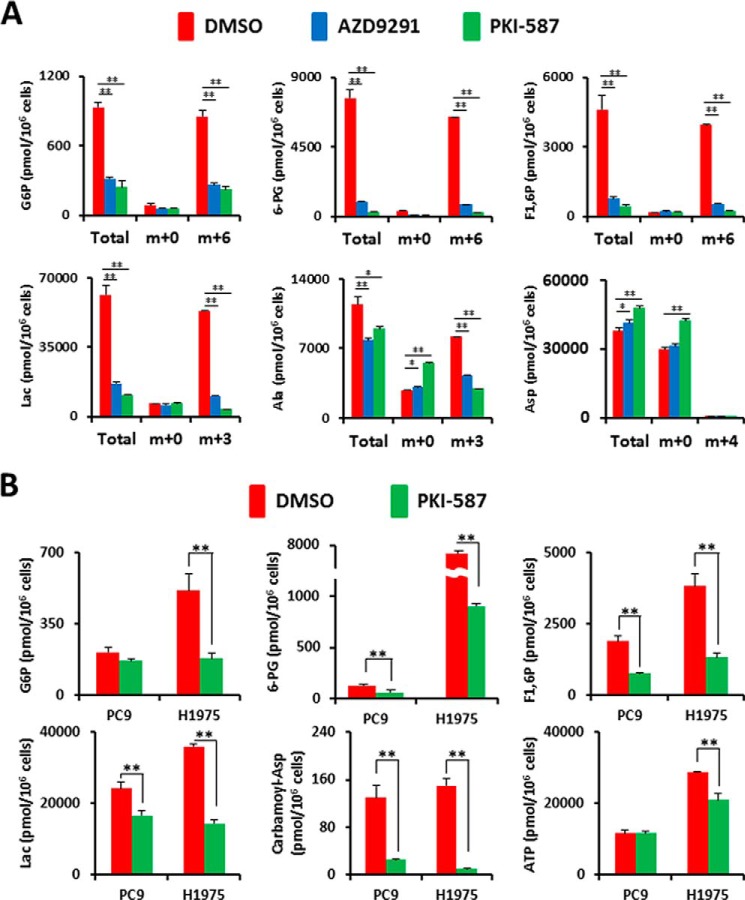
**Metabolomic profiling after inhibition of the PI3K/AKT/mTOR pathway.** Intracellular concentration (picomoles per million cells) of key metabolites involved in glycolysis and the PPP after the inhibition of EGFR signaling is shown. *Error bars* indicate mean ± S.D. (*n* = 3). Total metabolites were extracted with methanol from HCC827, PC9, or H1975 cells treated with DMSO (*red*) or inhibitors (*blue* or *green*) for 6 h. Representative metabolites such as Glc-6-P, 6-phosphogluconate (*6-PG*), fructose 1,6-bisphosphate (*F1,6P*), Lac, carbamoyl aspartic acid (*carbamoyl-Asp*), several amino acids, and ATP are shown. Others are listed in supplemental Table 1. *A*, flux profiling of ^13^C-labeled glycolytic and PPP metabolites. H1975 cells were grown in RPMI 1640 medium containing 11.1 mm [U-^13^C]glucose ([^13^C]Glc_6_) for 6 h in the presence of DMSO (*red bar*) or inhibitors (*blue* or *green bars*). Total Glc-6-P, [^13^C_0_]Glc-6-P (m+0), [^13^C_6_]Glc-6-P (m+6), total 6-PG, [^13^C_0_]6-PG (m+0) [^13^C_6_]6-PG (m+6), total F1,6P, [^13^C_0_]F1,6P (m+0), [^13^C_6_]F1,6P (m+6), total Lac, [^13^C_0_]Lac (m+0), [^13^C_3_]Lac (m+3), total Ala, [^13^C_0_]Ala (m+0), [^13^C_3_]Ala (m+3), total Asp, [^13^C_0_]Asp (m+0), and [^13^C_4_]Asp (m+4) are shown. **(B)** Static intracellular metabolites were quantitatively analyzed in PC-9 and H1975 cells treated with PI3K/mTOR inhibitor using capillary electrophoresis time-of-flight mass spectrometry (CE-TOFMS).

Our flux analysis indicated that glucose-derived lactate production could be reduced by inhibition of the PI3K/AKT/mTOR but not the RAS/MEK/MAPK pathway. We next used a metabolomics approach to quantify intracellular metabolites in LAD cells in the presence or absence of inhibitors. First, we tested the metabolic consequences of EGFR or PI3K/mTOR inhibition in H1975 cells given uniformly labeled [U-^13^C]glucose under our experimental conditions (Fig. 4*A*). Although the levels of metabolites at a steady state are the result of the balance between production and consumption, the use of the ^13^C label allows us to determine not only steady levels of metabolites but also the isotopomer and isotopologue distributions of metabolites derived from ^13^C-labeled glucose for the reconstruction of metabolic pathways (30, 31). We measured the metabolites derived from [^13^C]glucose in glycolysis and PPP such as glucose 6-phosphate (Glc-6-P), 6-phosphogluconate, and lactate (Lac) in the presence of DMSO, AZD9291, and PKI-587 (Fig. 4*A*). Repression of EGFR signaling in H1975 cells with AZD9291 resulted in a reduction of total and [13C]glucose-derived Glc-6-P, 6-phosphogluconate, and fructose 1,6-bisphosphate (F1,6P), which are early metabolites in glycolysis and PPP. The impact of PKI-587 on metabolites in H1975 cells was similar as that for TKIs, except for several metabolites such as amino acids (Fig. 4*A*). We also confirmed that the final product of glycolysis, total and [^13^C]lactate (m+3), were decreased after treatment with AZD9291 and PKI-587 (Fig. 4*A*).

To quantify the reduction of metabolite amount at a steady state, we extracted intracellular metabolites with methanol and analyzed them using CE-TOF/MS. Metabolome analysis revealed that the upstream metabolites in glycolysis and PPP were reduced after PKI-587 treatment for 6 h in both PC-9 and H1975 cells (Fig. 4*B* and supplemental Table 1). The carbamoyl-phosphate synthetase 2, aspartate transcarbamoylase, and dihydroorotase protein is required for the first step in the *de novo* synthesis of pyrimidines, and this protein is activated by mTOR via ribosomal protein S6 kinase 1 (S6K) (32). As expected, carbamoyl aspartic acid (carbamoyl-Asp) levels were decreased after PI3K/mTOR inhibitor treatment, as determined by metabolome analysis (Fig. 4*B*). In contrast, several amino acids were increased after inhibition of PI3K/AKT/mTOR signaling (supplemental Table 1). Moreover, the reduction of intermediate metabolites in glycolysis and PPP, decreased carbamoyl-Asp amount, and accumulating amino acids were reproduced in both H1975 cells treated with AZD9291 and HCC827 cells treated with PKI-587 (supplemental Table 1). Taken together, the metabolomics analysis indicates that the PI3K/AKT/mTOR signaling pathway is indispensable for aerobic glycolysis and *de novo* pyrimidine biosynthesis in *EGFR*-mutated LAD cells.

##### PI3K/AKT/mTOR Signaling Maintains the Membrane Localization of GLUT1

**FIGURE 5.**
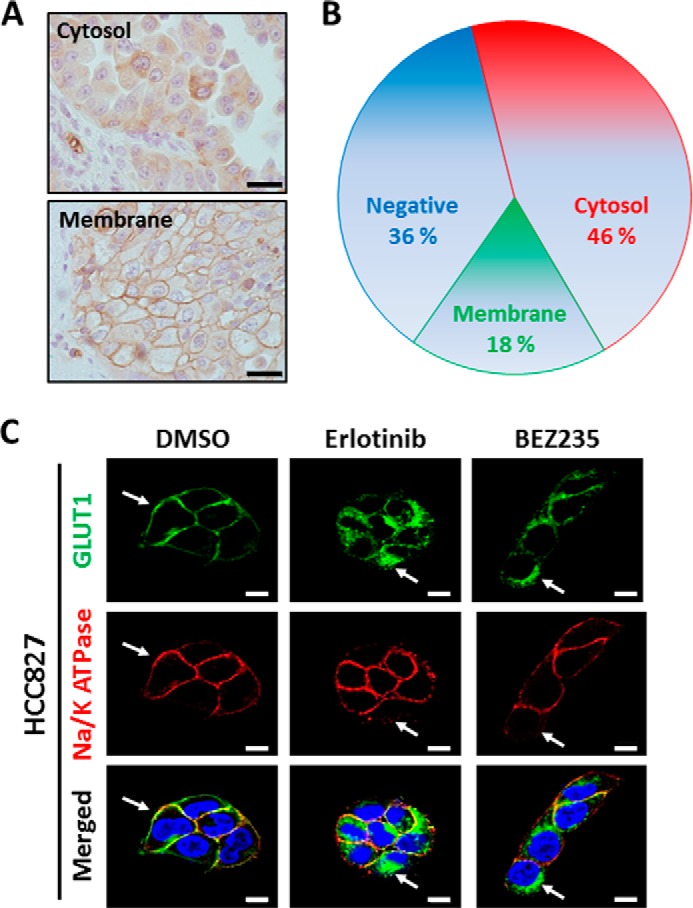
**PI3K/AKT/mTOR signaling maintains the membrane localization of GLUT1.** *A*, representative images from immunohistochemistry using GLUT1 antibody to identify cytosolic and membrane staining. *Scale bars* = 20 μm. *B*, pie chart summarizes the percentages of *EGFR*-mutant LAD tissue sections that are negative for GLUT1 protein (*blue*) or expressing GLUT1 predominantly in the cytoplasm (*red*) or plasma membrane (*green*). *C*, HCC827 cells stained with Alexa Fluor 488 (*green*, *GLUT1*), Alexa Fluor 594 (*red*, *Na/K ATPase*), and DAPI (*blue, nuclei*). Na/K ATPase was a positive control as a plasma membrane protein marker. *Scale bars* = 10 μm.

**FIGURE 6.**
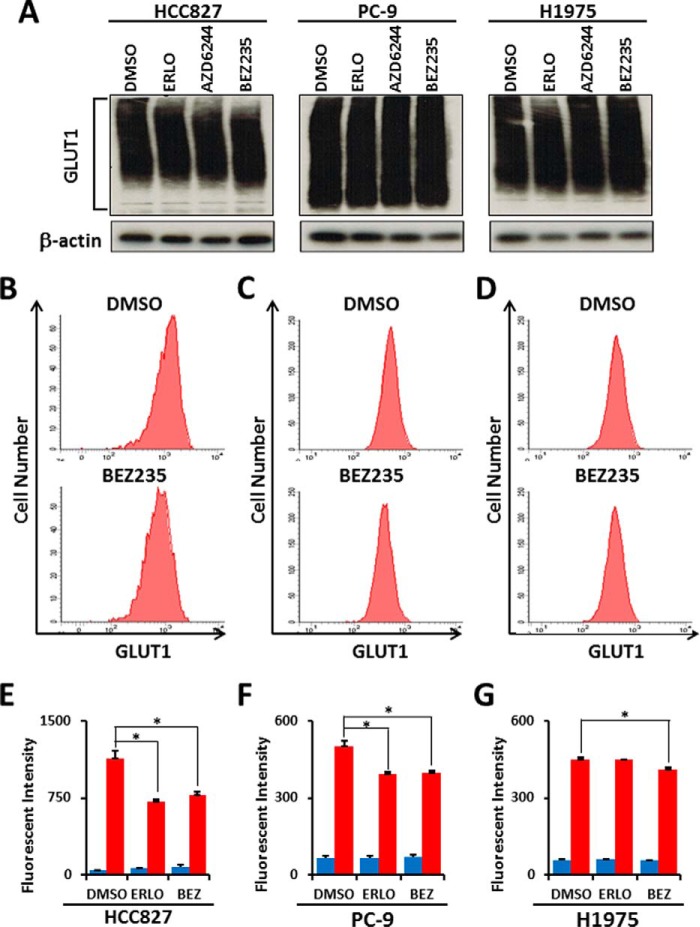
**Inhibition of the PI3K/AKT/mTOR pathway does not affect total GLUT1 protein expression but alters membrane-bound GLUT1 levels in *EGFR*-mutant LAD cells.** *A*, Western blot analysis showing GLUT1 and β-actin as a loading control in HCC827, PC-9, and H1975 cells treated with the indicated inhibitors. Equivalent amounts of proteins from whole cell lysates were subjected to WB analysis to detect total GLUT1 proteins. For flow cytometric analysis, LAD cells were treated with ERLO (1 μm), BEZ235 (1 μm) or DMSO as a control for 6 h. After fixation, cells were stained with a rabbit anti-GLUT1 antibody and FITC-conjugated anti-rabbit secondary antibody. *B—D*, representative flow cytometry plots of GLUT1 expression in HCC827 (*B*), PC-9 (*C*), and H1975 (*D*) cells treated with DMSO or BEZ235. *E—G*, mean fluorescence intensity for GLUT1 for HCC827 (*E*), PC-9 (*F*), and H1975 (*G*) cells. *BEZ*, BEZ235. *Blue bars* show background fluorescence with the IgG isotype control, whereas *red bars* indicate fluorescence staining results with anti-GLUT1 antibody. *Error bars* indicate mean ± *S.D.* (*n* = 3). *, *p* < 0.05 *versus* control by two-tailed Student's *t* test.

A comprehensive metabolomics analysis suggested that glucose transporter and hexokinase activities could be regulated by PI3K/AKT/mTOR signaling. Our previous genomics analysis of mRNA expression in LAD cell lines indicated a predominant up-regulation of the glucose transporter family member GLUT1 (*SLC2A1*) (33). To assess whether GLUT1 was expressed on the plasma membrane in *EGFR*-mutant LAD tissues, we performed a immunohistochemical analysis of 33 *EGFR*-mutant LAD cases. Representative immunohistochemistry evaluations of cytosolic or membrane GLUT1 positivity with anti-GLUT1 antibody are shown (Fig. 5*A*). We found that GLUT1 was predominantly localized in the cytosol (46%), with some at the cell membrane (18%) (Fig. 5*B*). Interestingly, 36% of cases were GLUT1-negative despite their *EGFR*-mutant status, suggesting that glucose transporters other than GLUT1 might be operating in such tumor tissues (Fig. 5*B*). We next examined the effects of PI3K/mTOR inhibition on membrane-bound GLUT1 in HCC827 cells by immunofluorescence (Fig. 5*C*). We observed a significant fraction of GLUT1 localized at the plasma membrane in HCC827 cells under normal culture conditions (Fig. 5*C*). Upon 6-h exposure to EGFR and PI3K/mTOR inhibitors, however, GLUT1 appeared as punctate structures distributed throughout the cell as well as in structures concentrated in the perinuclear region (Fig. 5*C*). On the other hand, localization of GLUT1 was unchanged after 6 h of treatment with the MEK inhibitor AZD6244 (data not shown). Western blot analysis showed that total GLUT1 was not decreased in HCC827, PC-9, and H1975 cells after 6-h treatment with any inhibitors (Fig. 6*A*). To determine the effects of erlotinib and BEZ235 on the membrane expression of glucose transporters in HCC827, PC-9, and H1975 cells, we quantitatively measured cell surface GLUT1 levels by flow cytometry (Fig. 6, *B–G*). Representative flow cytometry plots of GLUT1 expression in HCC827 (Fig. 6*B*), PC-9 (Fig. 6*C*) and H1975 (Fig. 6*D*) cells treated with DMSO or BEZ235 are shown. We observed a reduction of membrane-bound GLUT1 in the erlotinib-sensitive cell lines HCC827 (Fig. 6*E*) and PC-9 (Fig. 6*F*) after 6-h erlotinib treatment, whereas the expression of GLUT1 was unchanged in erlotinib-resistant H1975 cells (Fig. 6*G*). On the other hand, treatment with BEZ235 down-regulated membrane GLUT1 levels in all three cell lines (Fig. 6, *B–G*). These results suggest that the PI3K/AKT/mTOR signaling pathway is required to maintain GLUT1 at the plasma membrane in *EGFR*-mutant LAD cells.

##### Loss of GLUT1 Decreases Lactate Production and Cell Growth

**FIGURE 7.**
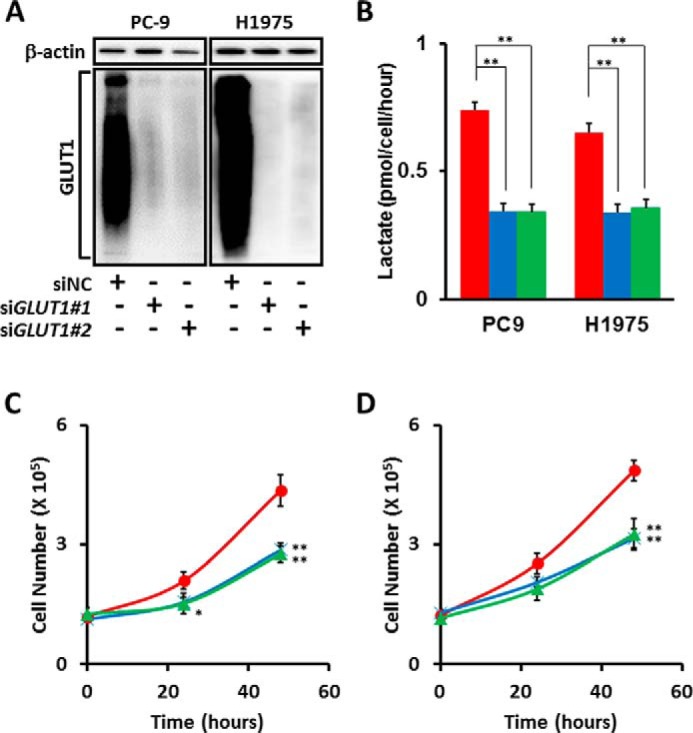
**Loss of GLUT1 in *EGFR*-mutant LAD cells decreases cellular proliferation and lactate production.** *A*, Western blot analysis of si*GLUT1*-treated LAD cells. siRNA targeting *GLUT1* (*SLC2A1*) successfully knocked down GLUT1 protein in PC-9 and H1975 cells, as confirmed by WB. The size signal for GLUT1 was widely distributed from 37–150 kDa. β-actin was used as a loading control for protein level. *B*, extracellular lactate production in PC-9 and H1975 cell lines transfected with siNC (*red*), si*GLUT1*#1 (*blue*), and si*GLUT1#2* (*green*). *C* and *D*, involvement of GLUT1 in cell growth. PC-9 (*C*) and H1975 (*D*) cells transfected with GLUT1 siRNAs were incubated for the indicated times. The data are shown as mean ± S.D. (*n* = 3). *, *p* < 0.05; **, *p* < 0.01 (Student's *t* test).

To further characterize the function of GLUT1 in LAD cells, we employed a genetic approach to repress *GLUT1* expression by RNAi. Western blot analyses revealed significant decreases in GLUT1 protein expression upon the introduction of two targeting RNAi constructs, si*GLUT1*#1 and si*GLUT1*#2, compared with a non-targeting control (siNC), in PC-9 and H1975 cells under normal culture conditions (Fig. 7*A*). To measure glycolytic activity in si*GLUT1*-transfected LAD cells, we quantified lactate in culture medium. Loss of GLUT1 significantly lowered the rate of lactate accumulation in the medium of LAD cell lines (Fig. 7*B*). Moreover, the number of PC-9 and H1975 cells decreased significantly 24 and 48 h after the introduction of si*GLUT1* (Fig. 7, *C* and *D*). Together, these results indicate that GLUT1 is important for glucose metabolism and the survival/proliferation of LAD cells and that the PI3K/AKT/mTOR signaling pathway appears to play a critical role in this process, likely by supporting the proper membrane localization of GLUT1 for optimal function in glycolysis.

## Discussion

In our previous paper, we showed that treatment with TKIs to EGFR, namely gefitinib and erlotinib, repressed aerobic glycolysis in *EGFR*-mutant LAD cells (11). Here we expanded upon those results by demonstrating that the PI3K/AKT/mTOR signaling pathway downstream of EGFR maintains aerobic glycolysis through GLUT1 function in *EGFR*-mutated LAD cells. Regulation of GLUT1 localization by PI3K/AKT/mTOR signaling is important for supporting glycolysis and the pentose phosphate pathway because loss of GLUT1 via suppression by siRNA to GLUT1 significantly lowered the rate of lactate accumulation and cellular growth of *EGFR*-mutant LAD cell lines. We conclude that signaling through the PI3K/AKT/mTOR pathway, but not the RAS/MEK/ERK pathway, is responsible for aerobic glycolysis and GLUT1 localization in *EGFR*-mutated LAD cells. Moreover, our metabolomic analysis revealed that PI3K/AKT/mTOR signaling maintains *de novo* pyrimidine synthesis and the amino acid profile in *EGFR*-mutated LAD cells.

Although resting T cells have low metabolic requirements and mainly use oxidative phosphorylation to generate ATP, activated T cells shift metabolic activity to aerobic glycolysis in a manner similar to tumor cells (34, 35). During T cell activation, several metabolic checkpoints have been suggested to influence the cell cycle, differentiation, cell fate, and immunological function (34, 35). A recent review theorized that metabolic checkpoints may exist to determine cell fate even in cancer cells because many factors that have been characterized as cell death regulators are known to modulate metabolic enzyme activity (36). Here we show that treatment of LAD cells with TKIs and PI3K/mTOR inhibitors diminishes the levels of metabolites in glycolysis and PPP, although it is still not clear which metabolite or metabolic enzyme is responsible for LAD cell survival. Our results suggest that one metabolic checkpoint in EGFR-mutated LAD cells could be glucose metabolism.

The molecular mechanism by which PI3K/AKT/mTOR signaling regulates glucose transport is still unclear. In adipocytes and skeletal muscle, insulin signaling and the PI3K/AKT pathway stimulate the translocation of intracellular GLUT4 to the cell surface to promote glucose uptake into cells (37). The insulin signaling pathway is regulated through AS160 (Akt substrate of 160 kDa) and Tbc1Ds to modulate Rab GTPase and through Rho GTPase TC10a to act on other targets (37). To test whether GLUT1 localization in *EGFR*-mutant LAD cells is regulated by a molecular mechanism similar to GLUT4, we examined the phosphorylation of AS160 proteins under our experimental conditions. Unexpectedly, phosphorylated AS160 protein was not changed 6 h after treatment with any tested inhibitors (data not shown). In addition, the role of intracellular GLUT1 is still unknown. Therefore, further investigation would be needed to characterize in greater detail the molecular mechanisms that control GLUT expression, activity, and translocation.

A previous study showed that newly synthesized GLUT4 accumulates within insulin-responsive compartments, whereas GLUT1 biosynthetically traffics to the plasma membrane in adipocytes (38, 39). After reaching the plasma membrane, GLUT1 undergoes endocytosis and localizes to recycling endosomes, where membrane proteins are transported for recycling back to the plasma membrane (40, 41). In this study, when EGFR and PI3K/AKT/mTOR signaling were inhibited, GLUT1 was predominantly found at intracellular compartments. Our data raise the possibility that PI3K/AKT/mTOR signaling plays an essential role in trafficking of GLUT1 from recycling endosomes and/or retention of GLUT1 at the plasma membrane. Further study will be required to understand the mechanism by which PI3K/AKT/mTOR signaling controls the intracellular dynamics of GLUT1.

A research report showed that tumor-associated mutant p53 (mutp53) stimulated the Warburg effect in cancer cells as a new mutp53 gain of function (42). Mutp53 did not change the expression of GLUT1 but promoted aerobic glycolysis by inducing GLUT1 translocation to the plasma membrane, which was mediated by activated RAS homolog gene family member A (RHOA) and the downstream effector Rho-associated, coiled-coil containing protein kinase 1 (ROCK). In our study, all three LAD cell lines possess homozygous mutp53 in that p.V218delV was identified in HCC827, p.R248Q in PC-9, and p.R273H in H1975 cells, as shown in public databases. A possible molecular mechanism is that either PI3K/AKT/mTOR signaling may regulate GLUT translocation by directly activating the RHOA/ROCK pathway or stimulate glycolysis through this mutp53 pathway. Another interesting study showed that 5′ AMP-activated protein kinase-dependent degradation of thioredoxin-interacting protein upon metabolic stress led to enhanced glucose uptake via GLUT1 (43). The PI3K/AKT/mTOR signaling pathway in *EGFR*-mutant LAD cells may be linked with the cellular redox status because the pentose phosphate pathway, which is a major source of NADPH in animal cells, was down-regulated after PI3K/mTOR inhibition. The molecular linkages between PI3K/AKT/mTOR signaling, oxidative stress, and GLUT1 are still unclear. Lee *et al.* (44) recently demonstrated that GLUT1 was phosphorylated on Ser-226 by PKC. This phosphorylation of Ser-226 was required for the rapid increase in glucose uptake and enhanced GLUT1 localization to the cell surface in endothelial cells. Additional experiments are required to determine whether PI3K/AKT/mTOR signaling controls glucose transport through the thioredoxin-interacting protein, mutp53, or the PKC pathway.

The application of metabolomics in oncology has focused on advances in both cancer imaging and therapy (15, 45, 46). A high rate of [^18^F]fluorodeoxyglucose uptake on positron emission tomography was significantly associated with a poor prognosis in lung cancer (47). From our work, we found that hyperactive glycolysis markedly increased lactate accumulation in lung tumor tissues (48). These results imply that targeting the PI3K/AKT/mTOR-GLUT axis could be a feasible chemotherapeutic option to control tumor progression. Indeed, compounds inhibiting the PI3K/AKT/mTOR pathway are currently in various stages of clinical development in oncology. However, the administration of inhibitors to the PI3K/AKT/mTOR pathway has been associated with metabolically adverse events, including hyperlipidemia and hyperglycemia (49–51). We showed that knockdown of *GLUT1* decreased cell growth in *EGFR*-mutant LAD cells but did not induce complete cell death. This may be due to the redundancy of other glucose transporters, such as GLUT3. Because the PI3K/AKT/mTOR pathway regulates many downstream effectors by cross-talking with various compensatory signaling pathways (51), the cell death induced by inhibition of PI3K/AKT/mTOR may be associated with several molecular mechanisms, including the internalization and degradation of GLUT1. Although agents directly inhibiting GLUT1 are in early-phase evaluations, preclinical studies have demonstrated that GLUT1 inhibitors led to diminished tumor growth *in vitro* and *in vivo* (52). Given that PI3K/mTOR inhibitors repress aerobic glycolysis in *EGFR*-mutated LAD cells, further studies are warranted to understand how cancer cell metabolism is regulated and to develop more effective therapeutic agents specifically targeted to the metabolic pathways that can limit cancer growth.

## Author Contributions

H. M. and K. T. conceived and coordinated the study and wrote the paper. M. T., S. M., and E. S. designed, performed, and analyzed the experiments shown in Figs. 1–3. H. M. designed, performed, and analyzed the experiments shown in Fig. 4. K. S., S. U., Y. O., and G. I. designed, performed, and analyzed the experiments shown in Figs. 5 and 6. M. T. designed, performed, and analyzed the experiments shown in Fig. 7. A. O., R. A., K. G., and H. E. contributed to the coordination, interpretation, and analysis of the data in this study. All authors reviewed the results and approved the final version of the manuscript.

## Supplementary Material

Supplemental Data

## Footnotes

2 The abbreviations used are:

LAD: lung adenocarcinoma
EGFR: epidermal growth factor receptor
mTOR: mammalian target of rapamycin
TKI: tyrosine kinase inhibitor
PPP: pentose phosphate pathway
ERLO: erlotinib
DMSO: dimethyl sulfoxide
ECAR: extracellular acidification rate
OCR: oxygen consumption rate
GLUT: glucose transporter
CE: capillary electrophoresis
WB: Western blot
Lac: lactate
F1,6P: fructose 1,6-bisphosphate.
