# Supplementary material for: Signaling through the Phosphatidylinositol 3-Kinase (PI3K)/Mammalian Target of Rapamycin (mTOR) Axis Is Responsible for Aerobic Glycolysis mediated by Glucose Transporter in Epidermal Growth Factor Receptor (EGFR)-mutated Lung Adenocarcinoma
Source: J Biol Chem. 2015 May 28;290(28):17495–504. doi: 10.1074/jbc.M115.660498 (PMC4498084; doi:10.1074/jbc.M115.660498)
Supplement: Supplemental Data [file supp_290_28_17495__index.html]

Signaling through the Phosphatidylinositol 3-Kinase (PI3K)/ Mammalian Target of Rapamycin (mTOR) Axis is Responsible for Aerobic Glycolysis mediated by Glucose Transporter in Epidermal Growth Factor Receptor (EGFR)-mutated Lung Adenocarcinoma — Signaling through the Phosphatidylinositol 3-Kinase (PI3K)/Mammalian Target of Rapamycin (mTOR) Axis Is Responsible for Aerobic Glycolysis mediated by Glucose Transporter in Epidermal Growth Factor Receptor (EGFR)-mutated Lung Adenocarcinoma — The PI3K/AKT/mTOR Pathway Regulates Aerobic Glycolysis — Supplemental Data 

# Signaling through the Phosphatidylinositol 3-Kinase (PI3K)/Mammalian Target of Rapamycin (mTOR) Axis Is Responsible for Aerobic Glycolysis mediated by Glucose Transporter in Epidermal Growth Factor Receptor (*EGFR*)-mutated Lung Adenocarcinoma

## Supplemental Data

- Supplementary Table 1 (.pdf, 64 KB) - Meatbolomic profile of quantified 116 major metabolites
