# Supplementary material for: Signaling through the Phosphatidylinositol 3-Kinase (PI3K)/Mammalian Target of Rapamycin (mTOR) Axis Is Responsible for Aerobic Glycolysis mediated by Glucose Transporter in Epidermal Growth Factor Receptor (EGFR)-mutated Lung Adenocarcinoma
Source: J Biol Chem. 2015 May 28;290(28):17495–504. doi: 10.1074/jbc.M115.660498 (PMC4498084; doi:10.1074/jbc.M115.660498)
Supplement: Supplemental Data [file supp_M115.660498_jbc.M115.660498-1.pdf]

Supplementary Table 1. Meatbolomic profile of quantified 116 major metabolites

ID	Compound name	Pathway Label	H1975_AZD9291 vs H1975_DM			1975_PKI-587 vs H1975_DMS			PC9_PKI-587 vs PC9_DMSO			HCC827_PKI-587 vs HCC827_DMSO		
			Ratio ¶	*p*-value ‖		Ratio ¶	*p*-value ‖		Ratio ¶	*p*-value ‖		Ratio ¶	*p*-value ‖	
A_0001	NAD^+	NAD+	1.0	0.481		0.8	0.065		1.0	0.973		0.9	0.065	
A_0002	cAMP	cAMP	0.7	0.225		0.9	0.281		0.7	0.094		0.9	0.059	
A_0003	cGMP	cGMP	N.A.	N.A.		N.A.	N.A.		N.A.	N.A.		N.A.	N.A.	
A_0004	NADH	NADH	0.5	0.015	*	0.4	0.003	**	0.7	0.028	*	0.8	0.116	
A_0005	Xanthine	Xanthine	0.8	0.335		0.7	0.017	*	0.9	0.771		0.9	0.589	
A_0006	ADP-ribose	ADP-Rib	0.7	0.127		0.7	0.014	*	0.9	0.489		1.0	0.879	
A_0007	Mevalonic acid	Mevalonic acid	N.A.	N.A.		N.A.	N.A.		N.A.	N.A.		N.A.	N.A.	
A_0008	UDP-glucose	UDP-Glc	1.0	0.738		1.0	0.962		0.9	0.536		0.8	0.038	*
A_0009	Uric acid	Uric acid	0.9	0.626		0.7	0.028	*	0.8	0.720		0.8	0.055	
A_0010	$NADP^+$	NADP+	0.5	9.0E-04	***	0.6	0.020	*	0.8	0.217		0.8	0.015	*
A_0011	IMP	IMP	1.6	0.009	**	0.8	0.138		0.9	0.269		1.2	0.058	
A_0012	Sedoheptulose 7-phosphate	S7P	N.A.	N.A.		N.A.	N.A.		N.A.	N.A.		N.A.	N.A.	
A_0013	Glucose 6-phosphate	G6P	0.3	0.005	**	0.4	0.021	*	0.8	0.104		0.9	0.401	
A_0014	Fructose 6-phosphate	F6P	0.11	N.A.		4.0	0.068		0.7	0.075		0.7	0.077	
A_0015	Fructose 1-phosphate	D-F1P	N.A.	N.A.		<1	N.A.		0.5	9.3E-04	***	<1	N.A.	
A_0016	Galactose 1-phosphate	Gal1P	0.7	0.335		0.9	0.152		1.1	0.487		0.9	0.583	
A_0017	Glucose 1-phosphate	G1P	0.02	N.A.		0.3	0.030	*	1.2	0.585		0.6	0.385	
A_0018	Acetoacetyl CoA	AAcCoA	N.A.	N.A.		N.A.	N.A.		N.A.	N.A.		N.A.	N.A.	
A_0019	Acetyl CoA	AcCoA	0.5	0.465		N.A.	N.A.		N.A.	N.A.		0.3	0.310	
A_0020	Folic acid	Folic acid	0.9	0.804		0.8	0.411		0.9	0.532		0.9	0.464	
A_0021	Ribose 5-phosphate	R5P	0.2	0.004	**	0.2	0.006	**	0.5	0.078		0.6	0.023	*
A_0022	CoA	CoA	0.9	0.464		0.7	0.046	*	0.7	0.213		0.8	0.123	
A_0023	Ribose 1-phosphate	R1P	0.5	0.267		N.A.	N.A.		N.A.	N.A.		0.3	7.5E-05	***
A_0024	Ribulose 5-phosphate	Ru5P	0.2	2.6E-04	***	0.4	0.064		0.7	0.149		0.6	0.020	*
A_0025	Xylulose 5-phosphate	X5P	<1	N.A.		<1	N.A.		0.10	N.A.		0.6	0.137	
A_0026	Erythrose 4-phosphate	E4P	0.3	N.A.		N.A.	N.A.		N.A.	N.A.		0.6	0.045	*
A_0027	HMG CoA	HMG-CoA	0.8	0.178		0.6	0.011	*	0.9	0.459		0.7	0.031	*
A_0028	Glyceraldehyde 3-phosphate	Glyceraldehyde 3-p	0.09	0.013	*	0.2	0.009	**	1.2	0.769		0.6	0.133	
A_0029	NADPH	NADPH	1.7	0.023	*	0.7	0.054		0.9	0.615		1.0	0.916	
A_0030	Malonyl CoA	Malonyl-CoA	0.4	0.075		N.A.	N.A.		N.A.	N.A.		0.3	0.052	
A_0031	Phosphocreatine	Phosphocreatine	0.9	0.264		0.8	0.058		0.8	0.181		0.8	0.062	
A_0032	XMP	XMP	0.9	0.775		0.7	0.123		1.0	0.967		0.6	0.010	**
A_0033	Dihydroxyacetone phosphate	DHAP	0.2	0.021	*	0.2	0.028	*	0.6	0.063		0.9	0.464	
A_0034	Adenylosuccinic acid	Succinyl AMP	1.2	0.030	*	0.7	0.044	*	1.0	0.548		1.2	0.555	
A_0035	Fructose 1,6-diphosphate	F1,6P	0.3	0.015	*	0.3	0.009	**	0.4	0.015	*	1.0	0.855	
A_0036	6-Phosphogluconic acid	6-PG	0.10	0.004	**	0.14	0.005	**	0.5	0.035	*	0.6	0.002	**
A_0037	*N*-Carbamoylaspartic acid	Carbamoyl-Asp	0.4	0.009	**	0.07	0.004	**	0.2	0.019	*	0.3	0.001	**
A_0038	PRPP	PRPP	0.6	0.037	*	0.2	0.007	**	0.7	0.254		0.5	0.066	
A_0039	2-Phosphoglyceric acid	2-PG	1.3	0.282		0.5	0.007	**	0.8	0.028	*	0.6	0.025	*
A_0040	2,3-Diphosphoglyceric acid	Diphosphoglycerate	0.2	0.014	*	0.3	0.012	*	0.7	0.056		0.7	0.033	*
A_0041	3-Phosphoglyceric acid	3-PG	1.2	0.523		0.5	0.016	*	0.8	0.117		0.6	0.036	*
A_0042	Phosphoenolpyruvic acid	PEP	1.5	0.195		0.4	0.010	*	0.6	0.053		0.5	0.040	*
A_0043	GMP	GMP	1.3	0.027	*	0.9	0.259		1.0	0.998		1.2	0.196	
A_0044	AMP	AMP	1.2	0.068		0.8	0.020	*	0.9	0.312		1.1	0.414	
A_0045	2-Oxoisovaleric acid	2-KIV	N.A.	N.A.		N.A.	N.A.		N.A.	N.A.		1.0	0.741	
A_0046	GDP	GDP	1.8	0.004	**	0.9	0.064		1.0	0.868		1.2	0.233	
A_0047	Lactic acid	Lactic acid	0.3	0.002	**	0.4	1.1E-04	***	0.7	0.009	**	0.7	0.021	*
A_0048	ADP	ADP	1.9	1.2E-05	***	0.8	0.062		0.9	0.274		1.1	0.217	
A_0049	GTP	GTP	0.9	0.016	*	0.7	0.021	*	1.0	0.881		1.0	0.883	
A_0050	Glyoxylate	Glyoxylate	N.A.	N.A.		N.A.	N.A.		N.A.	N.A.		N.A.	N.A.	
A_0051	ATP	ATP	0.8	0.013	*	0.7	0.028	*	1.0	0.994		1.0	0.356	
A_0052	Glycerol 3-phosphate	Glycerol 3-phospha	0.7	0.009	**	0.7	0.043	*	0.6	0.004	**	0.9	0.077	
A_0053	Glycolic acid	Glycolic acid	N.A.	N.A.		N.A.	N.A.		N.A.	N.A.		N.A.	N.A.	
A_0054	Pyruvic acid	Pyruvic acid	0.4	0.007	**	0.3	0.002	**	1.4	0.111		0.6	0.003	**
A_0055	*N*-Acetylglutamic acid	N-AcGlu	0.9	0.260		1.1	0.697		0.2	0.122		1.0	0.599	
A_0056	2-Hydroxyglutaric acid	2-Hydroxyglutaric a	0.5	9.3E-05	***	0.4	1.9E-04	***	0.5	0.020	*	0.5	9.6E-04	***
A_0057	Carbamoylphosphate	Carbamoyl-P	N.A.	N.A.		N.A.	N.A.		N.A.	N.A.		N.A.	N.A.	
A_0058	Succinic acid	Succinic acid	0.8	0.004	**	0.6	0.028	*	0.8	0.241		0.7	0.003	**
A_0059	Malic acid	Malic acid	0.5	8.0E-04	***	0.6	0.004	**	0.7	0.010	*	0.6	6.7E-04	***
A_0060	2-Oxoglutaric acid	2-OG	0.7	0.013	*	0.6	7.1E-04	***	0.6	0.025	*	0.6	0.007	**
A_0061	Fumaric acid	Fumaric acid	0.5	2.4E-05	***	0.6	0.008	**	0.7	0.007	**	0.6	3.3E-05	***
A_0062	Citric acid	Citric acid	0.8	0.003	**	0.7	0.009	**	1.0	0.938		0.7	0.035	*
A_0063	*cis*-Aconitic acid	cis-Aconitic acid	0.8	0.043	*	0.7	0.018	*	1.0	0.890		0.7	0.033	*
A_0064	Isocitric acid	Isocitric acid	0.9	0.365		0.8	0.068		1.1	0.665		0.8	0.463	
C_0001	Urea	Urea	0.8	0.018	*	0.8	0.034	*	0.8	0.053		0.9	0.285	
C_0002	Gly	Gly	1.2	0.047	*	1.4	0.007	**	1.6	0.002	**	1.8	8.5E-04	***
C_0003	Putrescine	Putrescine	0.6	0.001	**	0.5	0.001	**	0.9	0.211		0.8	0.033	*
C_0004	Sarcosine	Sarcosine	0.8	0.272		1.1	0.119		2.2	1.2E-04	***	1.4	0.312	
C_0005	β-Ala	b-Ala	1.0	0.647		0.6	0.094		1.8	0.611		0.9	0.323	
C_0006	Ala	Ala	0.8	0.068		0.9	0.070		0.5	0.004	**	1.7	9.0E-04	***
C_0007	*N*,*N*-Dimethylglycine	DMG	N.A.	N.A.		1.0	0.508		0.7	3.8E-04	***	N.A.	N.A.	
C_0008	γ-Aminobutyric acid	g-Aminobutyric acid	1.1	0.496		N.A.	N.A.		N.A.	N.A.		1.0	0.861	
C_0009	Choline	Choline	2.1	5.0E-04	***	11	2.1E-05	***	N.A.	N.A.		1.7	0.003	**
C_0010	Ser	Ser	0.7	0.004	**	1.3	0.001	**	2.2	2.2E-04	***	2.1	0.002	**
C_0011	Carnosine	Carnosine	1.4	0.111		0.8	0.117		0.9	0.401		0.9	0.763	
C_0012	Creatinine	Creatinine	0.8	0.047	*	0.8	0.071		0.8	0.127		0.9	0.344	
C_0013	Pro	Pro	0.9	0.053		1.8	0.001	**	2.1	1.4E-04	***	1.3	0.027	*
C_0014	Val	Val	1.1	0.413		1.7	4.4E-05	***	2.4	2.1E-04	***	2.2	3.0E-04	***
C_0015	Betaine	Betaine	N.A.	N.A.		7.2	0.043	*	N.A.	N.A.		1.1	0.726	
C_0016	Thr	Thr	0.9	0.066		1.6	0.007	**	2.3	0.004	**	2.0	0.001	**
C_0017	Homoserine	Homoserine	N.A.	N.A.		N.A.	N.A.		N.A.	N.A.		N.A.	N.A.	
C_0018	Betaine aldehyde	BTL	N.A.	N.A.		N.A.	N.A.		N.A.	N.A.		N.A.	N.A.	
C_0019	Cys	Cys	1.3	0.210		1.4	0.392		1<	N.A.		1.1	0.812	
C_0020	Hydroxyproline	Hydroxyproline	0.8	0.005	**	1.1	0.025	*	1.5	0.013	*	1.3	0.028	*
C_0021	Creatine	Creatine	1.1	0.196		0.9	0.378		0.5	0.002	**	1.0	0.613	
C_0022	Ile	Ile	1.0	0.556		1.4	8.3E-04	***	2.1	0.002	**	1.8	7.4E-04	***
C_0023	Leu	Leu	1.0	0.997		1.5	0.001	**	2.1	3.7E-04	***	1.9	3.2E-04	***
C_0024	Asn	Asn	0.8	0.001	**	1.2	0.022	*	2.1	6.5E-04	***	1.7	0.001	**
C_0025	Ornithine	Ornithine	1.1	0.483		1.2	0.068		1.4	0.146		2.1	0.029	*
C_0026	Asp	Asp	1.3	9.0E-04	***	1.2	0.051		1.8	0.002	**	1.3	0.004	**

C_0029	Hypoxanthine	Hypoxanthine	N.A.	N.A.		1<	N.A.		N.A.	N.A.		N.A.	N.A.	
C_0030	Spermidine	Spermidine	0.9	0.239		0.9	0.130		0.9	0.077		1.0	0.771	
C_0031	Gln	Gln	0.8	0.095		1.6	0.004	**	2.1	0.002	**	2.5	0.025	*
C_0032	Lys	Lys	1.1	0.521		2.0	0.001	**	1.8	0.017	*	2.3	0.021	*
C_0033	Glu	Glu	0.9	0.018	*	0.9	0.101		1.1	0.025	*	1.0	0.341	
C_0034	Met	Met	1.0	0.862		1.4	0.008	**	2.3	0.007	**	2.0	3.6E-04	***
C_0035	Guanine	Guanine	N.A.	N.A.		N.A.	N.A.		N.A.	N.A.		N.A.	N.A.	
C_0036	His	His	1.0	0.807		1.5	0.025	*	2.2	0.002	**	1.9	0.005	**
C_0037	Carnitine	Carnitine	0.6	0.015	*	1.1	0.651		N.A.	N.A.		1.0	0.706	
C_0038	Phe	Phe	1.0	0.606		1.6	1.9E-04	***	2.2	1.1E-04	***	1.9	5.7E-04	***
C_0039	Arg	Arg	0.9	0.124		1.4	0.003	**	1.5	0.018	*	1.4	0.059	
C_0040	Citrulline	Citrulline	0.8	0.070		1.2	0.047	*	1.6	0.033	*	1.6	0.049	*
C_0041	Tyr	Tyr	1.0	0.772		1.5	0.002	**	2.1	3.9E-06	***	1.9	2.0E-04	***
C_0042	*S*-Adenosylhomocysteine	SAH	0.8	N.A.		1.1	0.453		0.7	0.216		1.0	0.979	
C_0043	Spermine	Spermine	1.2	0.255		1.2	0.564		0.7	0.229		1.0	0.742	
C_0044	Trp	Trp	1.0	0.602		1.5	4.1E-04	***	2.1	2.0E-05	***	1.8	2.3E-04	***
C_0045	Cystathionine	Cystathionine	0.8	0.140		1.2	0.061		1.5	0.006	**	1.9	7.9E-04	***
C_0046	Adenosine	Adenosine	0.9	0.214		0.6	0.033	*	N.A.	N.A.		0.9	0.044	*
C_0047	Inosine	Inosine	0.6	N.A.		N.A.	N.A.		N.A.	N.A.		N.A.	N.A.	
C_0048	Guanosine	Guanosine	0.9	N.A.		1<	N.A.		N.A.	N.A.		1.4	N.A.	
C_0049	Argininosuccinic acid	ArgSuccinate	0.6	0.006	**	1.3	7.6E-04	***	1.4	0.039	*	1.0	0.848	
C_0050	Glutathione (GSSG)	GSSG	1.1	0.495		1.0	0.875		1.1	0.820		1.0	0.950	
C_0051	Glutathione (GSH)	GSH	0.9	0.086		0.8	0.045	*	1.0	0.976		0.9	0.129	
C_0052	*S*-Adenosylmethionine	SAM	0.9	0.046	*	1.1	0.010	*	1.1	0.465		1.0	0.648	
-	Adenylate Energy Charge	No Label	1.0	3.5E-04	***	1.0	0.084		1.0	0.190		1.0	0.220	
-	Total Adenylate	No Label	0.9	0.047	*	0.7	0.027	*	1.0	0.903		1.0	0.440	
-	Guanylate Energy Charge	No Label	1.0	2.2E-04	***	1.0	0.023	*	1.0	0.708		1.0	0.280	
-	Total Guanylate	No Label	0.9	0.051		0.7	0.022	*	1.0	0.895		1.0	0.720	
-	GSH/GSSG	No Label	0.8	0.159		0.8	0.305		1.0	0.877		0.9	0.569	
-	Total Glutathione	No Label	0.9	0.069		0.8	0.045	*	1.0	0.870		0.9	0.260	
-	NADPH/NADP+	No Label	3.2	0.018	*	1.1	0.531		1.2	0.132		1.2	0.239	
-	NADH/NAD+	No Label	0.5	0.007	**	0.5	6.4E-04	***	0.7	6.4E-04	***	0.9	0.536	
-	Lactate/Pyruvate	No Label	0.7	0.096		1.4	0.005	**	0.5	0.019	*	1.1	0.105	
-	Glycerol 3-phosphate/DHAP	No Label	3.7	0.003	**	4.6	0.002	**	1.0	0.966		1.0	0.957	
-	Total Amino Acids	No Label	1.0	0.147		1.1	0.048	*	1.9	3.9E-04	***	1.4	0.003	**
-	Total Essential Amino Acids	No Label	1.0	0.830		1.5	6.7E-05	***	2.2	2.0E-04	***	1.9	5.8E-04	***
-	Total Non-essential Amino Acids	No Label	1.0	0.110		1.1	0.076		1.8	3.6E-04	***	1.3	0.004	**
-	Total Glucogenic Amino Acids	No Label	1.0	0.130		1.1	0.054		1.8	4.2E-04	***	1.3	0.003	**
-	Total Ketogenic Amino Acids	No Label	1.0	0.672		1.5	2.8E-04	***	2.1	0.001	**	1.9	5.9E-04	***
-	Total BCAA	No Label	1.0	0.998		1.5	2.9E-04	***	2.1	2.9E-05	***	1.9	4.3E-04	***
-	Total Aromatic Amino Acids	No Label	1.0	0.674		1.6	3.5E-04	***	2.1	1.2E-05	***	1.9	2.1E-04	***
-	Fischer's Ratio	No Label	1.0	0.430		1.0	0.288		1.0	0.486		1.0	0.403	
-	Total Glu-related Amino Acids	No Label	0.9	0.011	*	1.0	0.521		1.7	2.6E-04	***	1.1	0.037	*
-	Total Pyr-related Amino Acids	No Label	1.0	0.863		1.3	0.014	*	2.0	7.1E-04	***	1.8	7.4E-04	***
-	Total Acetyl CoA-related Amino Acids	No Label	1.0	0.929		1.5	2.0E-04	***	2.1	5.9E-04	***	1.9	6.5E-04	***
-	Total Fumarate-related Amino Acids	No Label	1.0	0.687		1.6	3.1E-04	***	2.1	4.7E-05	***	1.9	2.3E-04	***
-	Total Succinyl CoA-related Amino Acids	No Label	1.0	0.977		1.5	2.0E-04	***	2.2	8.9E-06	***	1.9	3.2E-04	***
-	Total Oxaloacetate-related Amino Acids	No Label	1.1	0.008	**	1.2	0.038	*	2.0	0.002	**	1.4	0.002	**
-	Malate/Asp	No Label	0.4	0.001	**	0.5	0.004	**	0.4	0.005	**	0.5	0.002	**
-	Citrulline/Ornithine	No Label	0.8	0.013	*	1.0	0.657		1.1	0.372		0.8	0.014	*
-	Glu/2-Oxoglutarate	No Label	1.3	0.022	*	1.5	0.015	*	2.0	6.1E-05	***	1.9	0.002	**
-	G6P/R5P	No Label	1.6	0.068		2.0	0.033	*	1.6	0.035	*	1.5	0.047	*
-	SAM/SAH	No Label	1.0	N.A.		1.0	0.902		1.4	0.195		1.0	0.734	
-	Putrescine/Spermidine	No Label	0.7	0.042	*	0.6	0.007	**	1.0	0.774		0.8	0.075	

C: Cation mode, A: Anion mode
N.D.: Not Detected.
N.A.: Not Available.
Ration = Inhibitors/DMSO, n = 3.
[ll] Welch *t*-test, *p*-value (*<0.05, **<0.01, ***<0.001)
